# Application of Online Flow Cytometry for Early Biofouling Detection in Reverse Osmosis Membrane Systems

**DOI:** 10.3390/membranes14090185

**Published:** 2024-08-27

**Authors:** Laura Pulido Beltran, Johannes S. Vrouwenvelder, Nadia Farhat

**Affiliations:** 1 Environmental Science and Engineering, Biological and Environmental Science and Engineering (BESE) Division, King Abdullah University of Science and Technology, Thuwal 23955-6900, Saudi Arabia; laura.pulidobeltran@kaust.edu.sa (L.P.B.);; 2 Department of Biotechnology, Faculty of Applied Sciences, Delft University of Technology, Van der Maasweg 9, 2629 HZ Delft, The Netherlands

**Keywords:** seawater desalination, biofouling control, cleaning in place, biomass quantification

## Abstract

Biofouling poses a significant challenge to reverse osmosis (RO) membrane systems, necessitating timely detection for effective control. This study evaluated the efficacy of flow cytometry (FCM) for early biofilm detection in comparison to conventional system performance indicators. Feed channel pressure drop and total cell concentration in the Membrane Fouling Simulator (MFS) flowcell cross-flow outlet water were monitored over time as early biofouling indicators. The results demonstrated the potential of increased bacterial cell concentration in cross-flow outlet water as a reliable indicator of biofouling development on the membrane. Water outlet monitoring enabled faster biofouling detection compared to feed channel pressure drop. Membrane autopsy confirmed biofilm presence prior to the pressure drop increase, highlighting the advantage of early detection in implementing corrective measures. Timely intervention reduces operational costs and energy consumption in membrane-based processes.

## 1. Introduction

Fresh water is not evenly distributed worldwide, and due to increasing water demand, desalination processes have been developed and used to produce drinking water from brackish and seawater sources [1]. Water desalination through reverse osmosis (RO) has gained a lot of attention, and the application of RO has increased tremendously over the last decade [2]. RO produces high-quality water at an attractive cost [3]. One of the main concerns about this technology is membrane fouling [4,5,6] and specifically biofouling [7]. Biofouling is due to the excessive accumulation and growth of microorganisms forming a biofilm on the membrane surface [8], leading to a decline in membrane performance [4,9].

Several strategies have been studied and applied to control biofouling, such as improving pre-treatment performance and feed water quality to lower the substrate and bacterial load to the RO [10,11,12]. Other strategies include material and geometry modification [13,14,15] of the RO membranes or spacers that have been shown to either delay biofilm formation or lessen biofouling’s impact on performance. Membrane cleaning through chemical or mechanical processes has always been inevitable and applied to maintain plant performance [16,17]. Despite the progress in these strategies, biofouling continues to be a challenge in RO systems. Since microorganism adherence/growth on the membrane surface cannot be avoided [17], novel approaches to detect biofouling early have emerged [18,19].

In practice, detecting biofouling in membrane systems is commonly achieved through membrane autopsies or performance indicator changes [20]. An autopsy is an invasive technique that requires membrane destruction. Still, it allows for analysis specific to biomass presence and quantification (Adenosine Triphosphate, total cell concentration, among others) [21]. However, membrane performance indicators cannot directly be related to biofouling [22]. Instead, a decline in performance signals the deposition of material on the membrane surface [7] through the pressure drop increase, permeate flux, and salt rejection decline. A study conducted by Siebdrath et al. (2019) [7] found that among the three performance parameters mentioned previously, the pressure drop is the one that has the earliest and strongest increase when biofouling occurs on membranes, followed by a later decline in permeability and finally by salt rejection [7]. A new direction in membrane research is the application of ex situ, side-stream fouling detectors used as early warning sensors at full-scale and pilot plants [11,23,24]. The Membrane Fouling Simulator (MFS) is one ex situ fouling detector to monitor biological fouling in RO and NF. The MFS has the same hydraulic characteristics as a spiral wound membrane module and provides reproducible results. The MFS on its own can provide an effective early warning of biological fouling by monitoring feed channel pressure drop. The MFS, representing the first 0.20 m of the lead membrane module, will observe a pressure drop increase faster than the whole membrane installation.

Although performance indicators can monitor biofouling, it is detected later, when the biofilm has already formed, decreasing the effectiveness of applied cleaning methods [25]. Choosing an adequate biofilm monitoring technique is one key to mitigating biofouling in RO systems. For this reason, recent studies focused on novel approaches to detect biofouling at an early stage, enabling earlier corrective measures and anticipating better biofilm removal, therefore lessening the biofouling impact on membrane systems [22]. The focus of recent methods has been specifically to detect biofouling, rather than other fouling types, and particularly at an early stage and non-destructively. For example, O_2_ sensing optode luminescence imaging was used to assess early biofouling detection and quantification in an MFS simulating RO membrane system by Farhat et al. (2015) [18]. This non-destructive method measured a decline in oxygen concentration due to biofilm development, allowing the detection and quantification of biofouling on membrane surfaces at an early stage (before a pressure drop increase). Nevertheless, the application of O_2_ sensing optode luminescence imaging requires specific personnel expertise and equipment that are not easily found in practical settings. Likewise, RO modules cannot be monitored directly through this method. Therefore, this method must be performed with side-stream flowcells. Fluorogen-substrate cleavage is another non-destructive biofouling detection method, tested by Khan et al. (2019) [19]. This biosensor was developed using fluorogen-substrate cleavage that can detect fluorescence-based extracellular enzyme activity. The method was also tested using the MFS where a fluorogen substrate (MUF-Phosphate) was injected and a fluorescence analysis was performed through a spectrophotometer. Nonetheless, by using this technique, bacterial activity can be over- or underestimated due to the variability of bacterial enzyme production under stress conditions [26]. In addition, the production of extracellular enzymes is regulated not only by the bacterial growth phase but also by metabolic and physiological conditions [27].

Four main steps describe biofilm formation: (1) adsorption of organics on the membrane surface, (2) transport and attachment of microorganisms, (3) growth of attached microorganisms (biofilm maturation), and (4) detachment and recolonization [28,29]. During the last step, part of the biofilm is detached due to the shear forces caused by the feed water flowing through the system. Although detachment controls biofilm growth, it promotes biofilm dispersion through the membrane. When biofilm starts to grow, it also starts to be detached from the membrane. This detachment could have an impact on the bacterial total cell concentration (TCC) in the cross-flow water. Therefore, an increase in TCC in the cross-flow water can be an indication of biofouling development.

Several methods have been used to quantify TCC; however, flow cytometry (FCM) has gained more attention recently as the method is fast and reproducible. In this technique, cells are stained and detected by a flow cytometer through an emitted wavelength produced by the stained cells, which allows cell enumeration [30]. Likewise, new instruments have been developed to enable online FCM measurements [31]. This approach allows the monitoring of real-time bacterial cell concentration in water systems [32].

In this study, a flow cytometry-based approach, although restricted to the water phase, was used to measure bacterial cell concentration in MFS cross-flow water. The application of FCM to RO membrane biofouling assessment is appealing as measurements can be made while the membrane elements remain in service by analyzing the bacterial count of the feed, concentrate, and permeate streams [33]. In a previous study at a full-scale installation, Dixon et al. [33] showed that FCM has the potential to be used for non-destructive assessment of biofouling in RO elements and can be used to determine when the RO system is washing out or producing bacteria. This study aims to go steps further and evaluate the possibility of predicting biofilm growth at an earlier stage before performance indicators decline using FCM.

## 2. Materials and Methods

### 2.1. Experimental Setup

**Figure 1:**
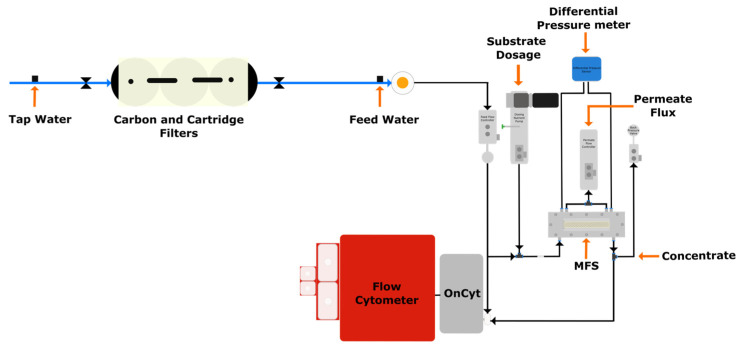
Experimental setup showing the sampling point for the online flow cytometry measurements.

Experiments were carried out in continuous filtration mode with Membrane Fouling Simulators (MFSs) to simulate biofilm growth on RO membranes and spacers [24] over time. Tap water produced at the King Abdullah University of Science and Technology Reverse Osmosis Desalination Plant was used to feed the system. An activated carbon filter and two cartridge filters (AC-SC-10-NL, Bluefilters, Senden, Germany) were used to remove any residual chlorine and particles in the tap water before feeding the system. The feed water was pumped to the MFS using a gear pump (EW-07002-25, Cole-Parmer, Vernon Hills, IL, USA), and the flow was controlled by a feed flow controller and a flow meter (mini CORI-FLOW™ M15, Bronkhorst, Vorden, The Netherlands). A substrate mixture of carbon, nitrogen, and phosphorous supplied at a ratio of 100:20:10 was pumped (D Series pump, Tuthill, Burr Ridge, IL, USA), controlled (mini CORI-FLOW™ M13, Bronkhorst, Vorden, The Netherlands), and mixed with the feed water before entering the MFS to promote biofilm growth of the tap water indigenous bacterial community. A back pressure controller valve (IN-PRESS P502CI, Bronkhorst, Vorden, The Netherlands) was used to regulate the outlet flow of the concentrate. The pressure drop increase over the feed channel was monitored with a differential pressure sensor (Deltabar PMD75, Endress + Hauser, Reinach, Switzerland). The online FCM was connected to the feed water before the substrate dosage and after the outlet of the concentrate. Both flows were controlled by a metering valve (Low Flow Metering Valve, 1/6 in. Tube Fitting, Swagelok, Koppel, PA, USA). Figure 1 shows the setup used for the experiments.

### 2.2. Experimental Design

The experiments were conducted in two sets of 4 independent MFSs each (8 in total), running in parallel (duplicates of each scenario). In each set, 2 MFSs were used as a control without substrate dosing that accelerates biofilm growth. The first set of experiments was stopped after an exponential increase in feed channel pressure drop was observed in the two MFSs where the substrate was dosed. At the end of the experiments, the pressure drop increase of the MFS with dosage was more than 50 mbar. The second set of experiments was stopped as soon as the total cell concentration in the feed channel outlet of the two MFSs dosed with substrate began to increase exponentially. When these experiments were stopped, no increase in pressure drop was observed.

### 2.3. Operating Conditions

The MFSs were operated with reverse osmosis membranes (FILMTECSW30HRLE-4040_DOW_USA) with dimensions of 200 mm × 40 mm and an active area of 8000 mm^2^. A 34 mil (864 µm) feed and permeate spacers (FILMTEC SW30HR LE-4040, DOW, Saginaw, MI, USA) with the same dimensions as the membranes were used. The feed flow was set at 20 L·h^−1^. The substrate dosage was pumped at 30 mL·h^−1^. The flow permeate was 60 mL·h^−1^.

A nutrient stock solution containing sodium acetate, sodium nitrate, and sodium phosphate in a mass ratio of C:N:P of 100:20:10 was used. The substrate solution increased the assimilable organic carbon concentration of the feed water by 200 μg C·L^−1^. Analytical-grade chemicals from Sigma Aldrich (Darmstadt, Germany) were purchased.

### 2.4. Fouling Monitoring

#### 2.4.1. Feed Channel Pressure Drop

Pressure drop measurements were recorded as an indicator of system performance for RO biofouling development monitoring, as feed channel pressure drop was shown to be the first and most strongly impacted performance indicator [7]. The average initial pressure drop of all the MFSs was 21 ± 3 mbar.

#### 2.4.2. Bacterial Cell Concentration in Water Using Online Flow Cytometry

An OnCyt robot (OnCyt, Otelfingen, Switzerland) was connected to a BD Accuri C6 plus flow cytometer (BD Accuri Cytometer, Aalst, Belgium) which uses a 50 mW laser to produce a fixed emission of 488 nm wavelength. Two of the four emission detectors were selected (FL1 = 533 ± 30 nm and FL3 > 670 nm) to collect the fluorescence signals. Likewise, the two scatter detectors (forward and sideward scatters) were used to obtain light intensities. To measure the total bacterial cell concentration using the SYBR green signals, an electronic gating was selected following the procedure described by [34].

The Oncyt robot was connected to the system to measure the feed water and outlet concentrate total cell concentration over time. The equipment can automatically take a sample, stain, and incubate every ten minutes, resulting in a total of six samples in one hour. The OnCyt robot automatically moves the samples to the flow cytometer (BD Biosciences, Aalst, Belgium) to carry out the measurements. The script used for the OnCyt equipment allowed for acquiring, staining, incubating, and measuring a 66 µL sample of each flow line every 20 min. Three measurements per hour from each flow line were taken until the end of the experiments. Each sample was stained by the OnCyt robot with a 2X SYBR green stain solution (prepared from a 10,000X concentration stock solution (Molecular Probes, Eugene, OR, USA)), in a TRIS buffer solution (10 mM, pH 8) containing sodium thiosulfate (Honeywell, Offenbach am Main, Germany (50 mM)). The final concentration of SYBR green in the sample was 1X. The equipment incubated each sample for 10 min at 37 °C before conducting the measurement. The OnCyt robot carried out cleaning after each measurement with hypochlorite (1% active chlorine; Honeywell, Germany), sodium thiosulfate solution (100 mM), and ultrapure water.

All the data were stored in the computer and then analyzed with the Cyplot software v3.12 (Oncyt, Otelfingen, Switzerland). The data from each experiment, the gates, and the plot setup were uploaded to the program. The data were compiled and plotted in Origin Pro data analysis software.

### 2.5. Biomass Quantification on the Membrane

#### 2.5.1. Total Bacterial Cell Count

Total bacterial cell counts (TCCs) in the biofilm were performed by flow cytometry, following the protocol reported by Neu et al. (2019) [35]. Coupons of 4 × 2 cm^2^ of the biofouled membrane and spacer were cut from the MFS. The coupons were then placed in a capped tube with 40 mL ultrapure water. The sample tubes were submerged in ice and sonicated for 30 s (Q700CA Sonicator, Qsonica, Newtown, CT, USA). Then, 700 µL from each sample was stained with 7 µL of a 100X SYBR green solution (prepared from a 10,000X concentration stock solution (Molecular Probes, Eugene, OR, USA)) and incubated for 10 min at 37 °C. From this mixture, 200 µL of sample was transferred to a 96-well plate to carry out the measurements.

The biofilm TCC measurements were performed using a BD Accuri C6 flow cytometer (BD Accuri Cytometer, Belgium) in a manual mode similar to the equipment described earlier. The data obtained were processed using the BD Accuri CFlow^®^ software (Aalst, Belgium). One replicate was carried out for each sample.

#### 2.5.2. Adenosine Triphosphate

The Adenosine Triphosphate (ATP) present in the biofilm was measured using a luminometer (Celsis Advance Luminometer, Charles River Laboratories, Inc., Wilmington, MA, USA). Similar to the TCC, the biofouled membrane and spacer were cut into coupons of 4 × 2 cm^2^, and then they were deposited in 50 mL centrifuge tubes with 40 mL of sterile tap water. Next, the tubes were submerged in ice and sonicated for 30 s (Q700CA Sonicator). After obtaining a homogeneous liquid, duplicates of 50 µL of the sample were taken and placed in cuvettes to measure the ATP in the luminometer.

For each sample, the equipment uses 200 µL of LuminEx (Charles River Laboratories, Wilmington, MA, USA) to release the ATP inside the cells and 100 µL of LuminATE-HS (Charles River Laboratories, Wilmington, MA, USA) to burn the ATP and produce light. This bioluminescence reaction emits light of 560 nm wavelength which is measured in Relative Light Units (RLUs) by the Celsis Advance Luminometer [36]. Then, a calibration curve that established a relation between RLUs and pg ATP/mL values was used to obtain the concentration values (pg/mL) from the RLUs of the samples.

## 3. Results

### 3.1. Can an Increase in Bacterial Cell Concentration in the Outlet Water Be Observed before the Pressure Drop Increases?

#### 3.1.1. Online Measurements: Pressure Drop Versus Bacterial Cell Concentration

**Figure 2:**
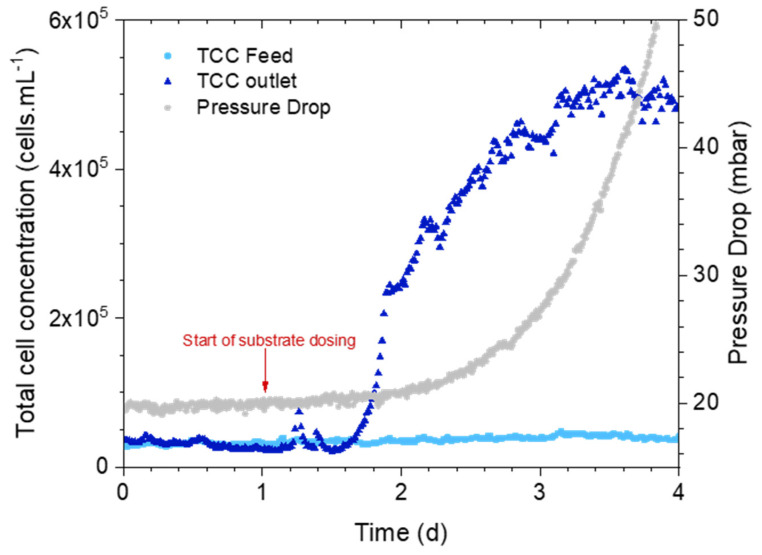
Feed channel pressure drop (mbar) and total cell concentration (TCC) (cells/mL) over time in the MFS+S inlet and outlet.

The first set of experiments was conducted to investigate whether the bacterial cell concentration parameter of the cross-flow water can signal biofilm development in the MFS. Figure 2 shows the average pressure drop and TCC of the inlet and outlet of the MFSs fed with substrate. The TCC measurement acquiring was started before the substrate dosage to establish a baseline for the inlet and outlet cross-flow concentration. On day one, the substrate dosage was started. The concentration of cells in the feed water was stable during the whole experiment, while the concentration in the outlet water started to increase around 12 h after substrate dosing. There were a few higher bacterial cell concentration measurements after the start of substrate dosing which may be attributed to any bacterial cells from the substrate dosing tube. However, these outliers did not affect the overall trend of the bacterial cell concentration measurement in time. Figure 2 shows that the pressure drop had no major increase when the TCC of the outlet began its exponential trend. The pressure drop showed an increase after 2 days when the TCC of the outlet had already increased more than 200%.

#### 3.1.2. Biofilm Characterization

**Figure 3:**
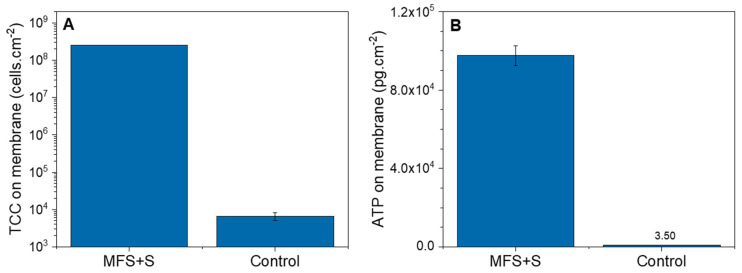
Biofilms: (**A**) total cell concentration (cells.cm^−2^) and (**B**) Adenosine Triphosphate (pg.cm^−2^) of the MFS+S membrane and control membrane obtained from the autopsy (after 3 days of substrate dosing).

The average biofilm TCC and ATP concentrations obtained from the autopsy of the membranes dosed with substrate were 2.55 × 10^8^ ± 1.6 × 10^6^ cells.cm^−2^ and 9.76 × 10^4^ ± 5.0 × 10^3^ pg.cm^−2^, respectively (Figure 3). Compared to the control membrane, where no substrate was dosed (6.6 × 10^3^ ± 1.6 × 10^3^ cells.cm^−2^ and 3.5 ± 0.1 pg.cm^−2^, respectively), the TCC and ATP concentrations were significantly higher. The autopsy confirmed the presence of a biofilm in the MFSs dosed with substrate as detected by the online TCC measurement of the outlet cross-flow water and the pressure drop.

### 3.2. How Early Is the Cell Concentration Increase Detected?

#### 3.2.1. Online Measurements: Pressure Drop Versus Bacterial Cell Concentration

**Figure 4:**
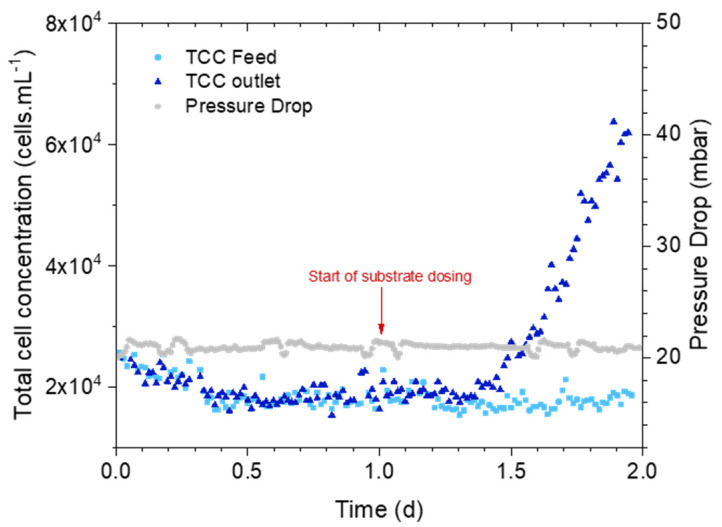
Feed channel pressure drop (mbar) and total cell concentration (cells.mL^−1^) over time in the MFS+S inlet and outlet water.

The second set of experiments focused on how early an increase in the outlet TCC is detected and the amount of biomass that can be detected through an autopsy. Figure 4 shows the average TCC and pressure drop of the MFSs dosed with the substrate. The inlet and outlet cross-flow TCC were stable before starting the substrate dosage. Similarly, 12 h after starting the substrate dosing, the concentration of cells in the outlet flow showed an exponential increase while the feed flow did not show any significant change. When the MFS was stopped, the TCC of the outlet was about three times more than the feed flow. During the whole experiment, there was no significant change in the pressure drop.

#### 3.2.2. Biofilm Characterization

**Figure 5:**
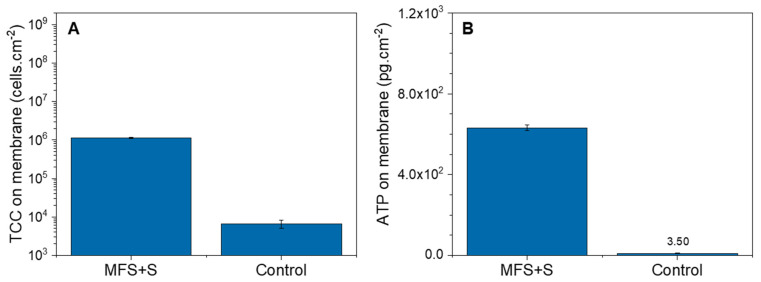
Biofilms: (**A**) total cell concentration (cells.cm^−2^) and (**B**) Adenosine Triphosphate (pg.cm^−2^) of the MFS+S membrane and control membrane obtained from the autopsy (after 1 day of substrate dosing).

The autopsy of the MFSs dosed with biodegradable substrate confirmed the presence of the biofilm. Figure 5 shows the average biofilm TCC and ATP concentrations from the MFSs with substrate dosage. In this experiment, both concentrations were higher than the control MFS with no substrate dosage but lower than the ones obtained in the previous set of experiments with dosage (1.1 × 10^6^ ± 3.3 × 10^4^ cells.cm^−2^ and 6.3 × 10^2^ ± 1.4 × 10^1^ pg.cm^−2^, respectively). 

## 4. Discussion

### 4.1. Biofilm Detection in Membrane Systems: Total Cell Concentration in the Outlet as an Indication of Biofilm Growth

Improvements to RO desalination membrane systems addressing pre-treatment, fouling, and energy requirements are crucial for lowering the cost of water production [37,38,39]. Biofouling is one of the major challenges of operating membrane systems [7,40], so the focus has been directed toward developing techniques to control it. Current methods to determine biofilm growth on the membrane surface are indirect or destructive [18]. Usually, these methods detect biofilm at a much later, more mature stage [19]; therefore, the efficiency of the corrective measurements could be reduced and this could lead to an increase in operational costs.

Understanding how biofilm is formed on the membrane surface is essential to develop methods to detect it early. Biofilm development starts with bacterial attachment followed by growth and later detachment and dispersion [28,41]. During the detachment stage, clusters of cells are separated from the biofilm due to shear forces applied by the water flow or changes in the clusters’ structure. Then, during the dispersion stage, clusters that enter into the bulk can either reattach on the membrane surface or remain in the bulk liquid. Results from this study showed that the total cell concentration in the concentrate water flow exponentially increased and reached a stationary phase at 3.5 days (Figure 2). Cells and cell clusters detached from the membrane surface and remained in the water [28]. An increase in the pressure drop indicated that a biofilm layer was already formed on the membrane surface, affecting the performance. The trend of TCC and pressure drop development could suggest an established biofilm since a plateau phase can occur during detachment due to nutrient limitation or shear forces [28]. The exponential increase in the released number of bacterial cells and clusters (biofilm formation) started at 1.5 days (12 h after the start of substrate dosing) and ended approximately at 3.5 days (Figure 2). However, the pressure drop started to increase only 1 day after the start of substrate dosing. The exponential increase in the released number of bacterial cells and clusters started within 12 h of nutrient dosage without any change in pressure drop. This indicated that an increase in cell concentration in the water was able to signal biofilm formation early and that pressure drop may not be the best parameter to detect biofilm formation at an early stage.

### 4.2. Accelerated Biofilm Development Timescale Translated to Reality

In reverse osmosis systems, biofilm formation on the membrane can occur within the first two weeks of operation and be fully developed after one or several months depending on the feed stream quality [42]. However, performance parameter decline, such as pressure drop, will not necessarily occur immediately. Vrouwenvelder et al. (2011) [22] performed a study at a full-scale membrane plant in which the normalized pressure drop (NPD) increase was <10% in the first 2.5 years of operation with an assimilable organic carbon (AOC) concentration of the feed water of <10 µg C L^−1^. After a change in the feed water quality with an AOC of 90 µg C L^−1^, an increase of 90% in NPD was noticed. Accelerated biofilm development experiments, such as the experiments conducted for this study, show how the membrane performance could be affected by biofouling not only over years, but within days or weeks. In this study, by increasing the feed tap water concentration to 200 µg C L^−1^, the biofilm development occurred in less than 5 days. This shows the impact of the quality of the feed stream on RO membrane performance.

Using online monitoring of the total cell concentration of the feed and concentrate streams would help detect biofilm formation at early stages. It would be interesting to assess whether there are differences in the TCC measured in the feed and concentrate streams directly from the membrane module and from the streams of an MFS fed with the same feed stream that enters the membrane modules (without any additional substrate dosage) and the concentrate stream of the MFSs.

### 4.3. Early Detection of Biofilm on Membrane Systems

An early non-destructive method to detect the biofouling development on membrane surfaces will allow for implementing timely solutions. Better biofilm removal from the membrane surface is anticipated when cleaning protocols are applied before biofilm layers become thicker and denser over time and difficult to remove [43]. An early detection of the formed biofilm was obtained during this study by monitoring the total cell concentration in the concentrate water flow. An exponential increase in total cell concentration after nutrient dosage was detected (Figure 4), and this trend continued before any change in the pressure drop. According to Flemming (2008, 2014) [28,41], during the second stage of biofilm formation, cells start to multiply and form clusters. During this stage, initial clusters are also exposed to shear forces produced by the water flow. As the biofilm forms and the adhesion among cells builds, some of the clusters or cells from the clusters may move into the water flow due to the shear, resulting in an increase in TCC in the concentrate flow as seen during the 12 h of the exponential phase in this study. Low levels of ATP and TCC on the membrane that were autopsied before any change in pressure drop (Figure 5) confirm an early stage of the formation of the biofilm. The ATP (6 × 10^2^ pg.cm^−2^) and TCC (10^6^ cells.cm^−2^) concentrations on the membrane were lower than the concentrations reported in 15 full-scale installations [44] at 10–1000% normalized pressure drop increase, confirming the FCM’s ability of earlier detection than the occurrence of pressure drop.

To prevent biofilm development to the mature stage on the membrane surface, cleaning should be applied as soon as the early stage of biofilm formation is detected to enhance biofilm removal [45,46]. Earlier cleaning might lead to higher cleaning frequency to control biofouling. Applying more frequent chemical cleanings can increase operational costs, affect membrane integrity and lifetime, and produce unwanted environmental consequences [47]. For that reason, there is a need to find novel cost-effective cleaning methods that preserve membrane integrity and are environmentally friendly. Recent studies have focused on green chemicals, such as urea [48], and on physical cleaning, such as using CO_2_/microbubbles and backwashing, among others [49,50], necessitating thorough investigation.

## 5. Conclusions

This study aimed to predict biofilm growth in its early stages before traditional performance indicators declined. Feed channel pressure drop and total cell concentration in the MFS cross-flow outlet water were monitored as potential early biofouling indicators. The MFSs were stopped according to the selected indicator for each MFS. Biofilm biomass was quantified using ATP and TCC analyses.

The results demonstrated that monitoring bacterial cell concentrations in effluent water effectively predicts biofilm development earlier than traditional pressure drop indicators. Membrane autopsy confirmed biofilm presence before a measurable increase in feed channel pressure drop. This early detection enables timely intervention, reducing operational costs and energy consumption in membrane processes.

## Data Availability

The original contributions presented in the study are included in the article, further inquiries can be directed to the corresponding author.
